# Developmental Markers Expressed in Neocortical Layers Are Differentially Exhibited in Olfactory Cortex

**DOI:** 10.1371/journal.pone.0138541

**Published:** 2015-09-25

**Authors:** Peter C. Brunjes, Stephen K. Osterberg

**Affiliations:** Department Psychology, University of Virginia, Charlottesville, Virginia, 22904, United States of America; School of Biomedical Sciences, The University of Queensland, AUSTRALIA

## Abstract

Neurons in the cerebral cortex stratify on the basis of their time of origin, axonal terminations and the molecular identities assigned during early development. Olfactory cortices share many feature with the neocortex, including clear lamination and similar cell types. The present study demonstrates that the markers differentially expressed in the projection neurons of the cerebral cortex are also found in olfactory areas. Three of the four regions examined (pars principalis of the anterior olfactory nucleus: AONpP, anterior and posterior piriform cortices: APC, PPC, and the olfactory tubercle) expressed transcription factors found in deep or superficial neurons in the developing neocortex, though large differences were found between areas. For example, while the AONpP, APC and PPC all broadly expressed the deep cortical marker CTIP2, NOR1 (NR4a3) levels were higher in AONpP and DAARP-32 was more prevalent in the APC and PPC. Similar findings were encountered for superficial cortical markers: all three regions broadly expressed CUX1, but CART was only observed in the APC and PPC. Furthermore, regional variations were observed even within single structures (e.g., NOR1 was found primarily in in the dorsal region of AONpP and CART expression was observed in a discrete band in the middle of layer 2 of both the APC and PPC). Experiments using the mitotic marker EDU verified that the olfactory cortices and neocortex share similar patterns of neuronal production: olfactory cells that express markers found in the deep neocortex are produced earlier than those that express superficial makers. Projection neurons were filled by retrograde tracers injected into the olfactory bulb to see if olfactory neurons with deep and superficial markers had different axonal targets. Unlike the cerebral cortex, no specificity was observed: neurons with each of the transcription factors examined were found to be labelled. Together the results indicate that olfactory cortices are complex: they differ from each other and each is formed from a variable mosaic of neurons. The results suggest that the olfactory cortices are not merely a remnant architype of the primordial forebrain but varied and independent regions.

## Introduction

The development of the cerebral cortex has been actively investigated for decades. The deep ventricular and subventricular layers of the early telencephalon produce several kinds of progenitor cells. The earliest neurons arising from these precursors come to reside within a structure known as the preplate that emerges by about embryonic day 10.5 in the mouse. About a day later the cortical plate begins to form within the preplate; subsequent maturation yields the six layers of the mature structure. The production of projection neurons (pyramidal cells) destined for the neocortex follows a strict sequence over a period of about 6 days in mice. Neurons occupying the deepest layers are formed first with successive divisions populating increasingly superficial layers. Each neocortical layer has characteristic neuronal types. For example, the deepest layer, layer 6, houses most of the corticofugal cells forming feedback connections to thalamic nuclei. Most of the neurons projecting to a variety of non-thalamic targets, including the pons, superior colliculus and the spinal cord, are found in layer 5. Intra-cortical projection neurons preferentially reside in the superficial layers 2–3 [1–6].

**Fig 1:**
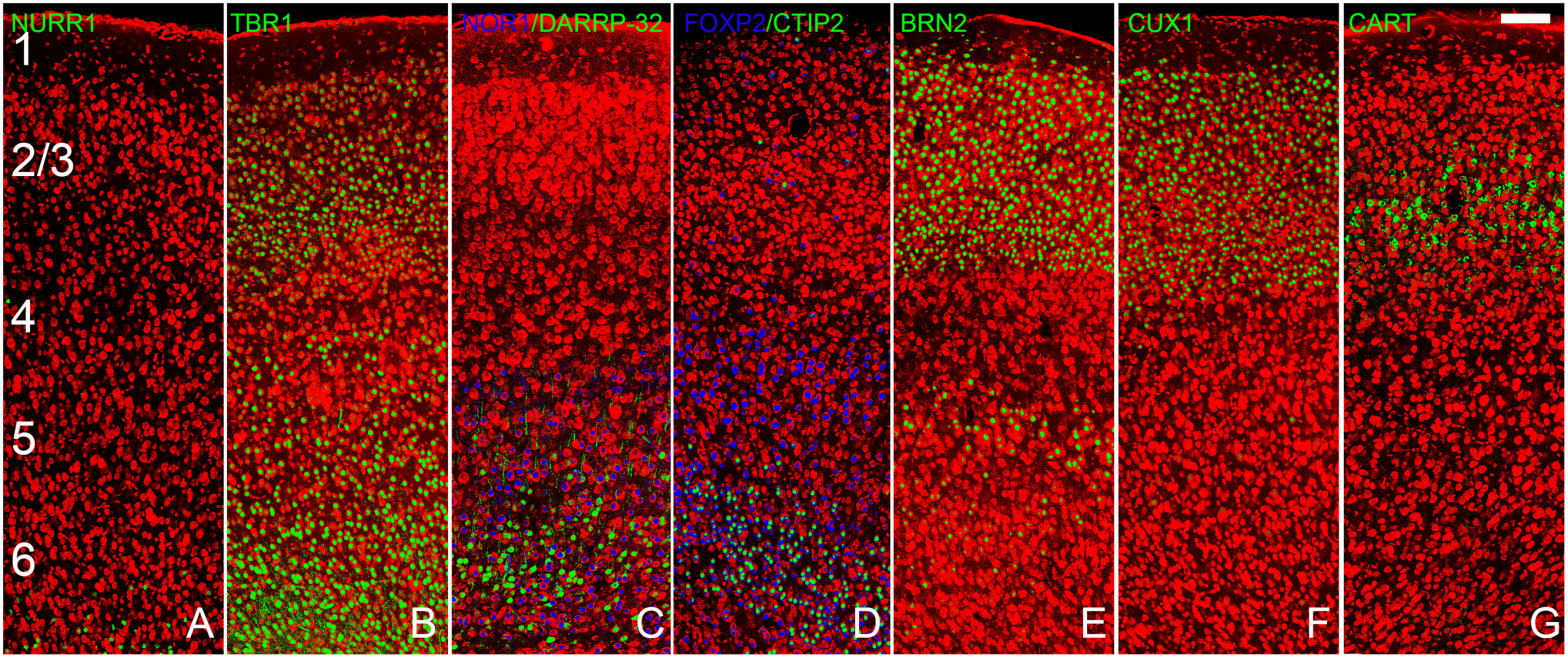
Staining patterns for each of the probes used in the present study in the frontal neocortex. Panel A shows staining for NURR1 labeled cells in the preplate; the deepest region of the cortex. TBR1 (panel B) labeled all projection neurons in the cortex, including those found in layers 2, 3, 5 and 6. Panels c and d depict four markers for deep (5 and 6) layers: NOR1 (Nr4A3), DAARP-32; FOXP2 and CTIP2. BRN2 identified cells in layers 2 and 3 as well as a small number of cells in layer 5 (panel E). The superficial markers used included CUX1 (Layer 2–4, Panel F) and CART (Layer 3, Panel G). Scale bar = 200μm. Numbers in panel A delineate cortical layers. See text for details.

The progressive changes in the precursor cells that allow the formation of these different classes of projection neurons have been extensively examined. Dozens of transcription factors have been identified that are characteristically expressed by both germinal cells and by neurons residing in specific cortical layers (Fig 1; [5, 7–10]). For example, some preplate progenitors and neurons express Nurr1 (also known as NR4A2 [11–12]). Most glutamatergic pyramidal cells express TBR1 [13–17]. Early-born cells that become neurons residing in layers 5 and 6 express markers such as FOXP2 [18], CTIP2 [19], DARPP-32 [20, 21] and NOR1 (also known as NR4A3 [7]). BRN2 labeling is associated with neurons found in layers 2/3 and a small cohort of cells in layer 5 [1, 22, 23]. Other markers have been discovered that are associated primarily with cortico-cortical projection neurons found in superficial layers (e.g., CUX1 in layers 2–4 [24] and CART in layer 3 [25, 26]). Thus, different populations of cortical neurons can be identified by their time of origin, laminar location, projection pattern, and molecular signatures.

**Fig 2:**
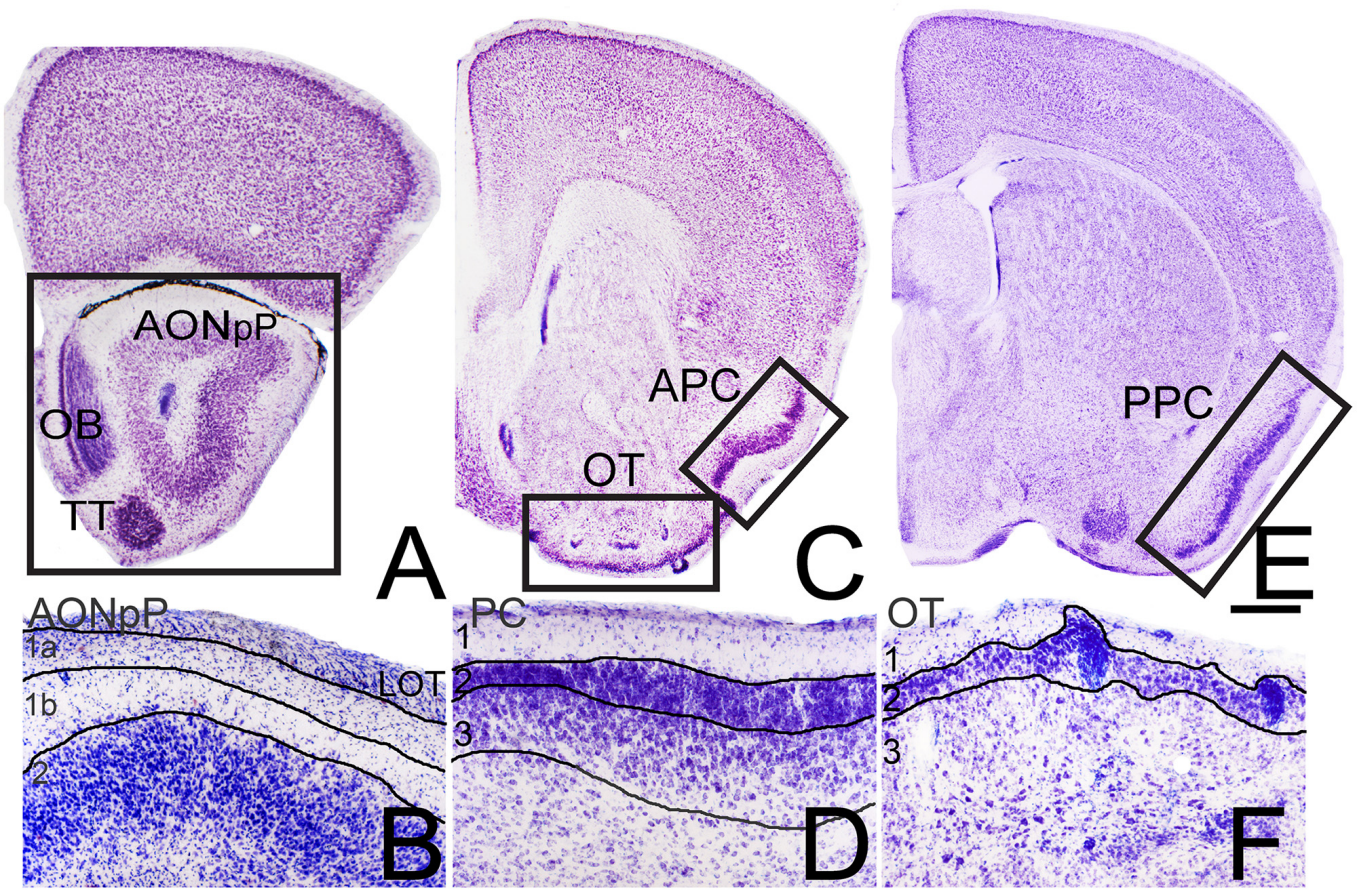
Nissl section depicting the regions studied. The low power micrographs in panels A, C and E show the regions from which sections were selected to examine pars principalis of the anterior olfactory nucleus (AONpP, Panel A), anterior piriform cortex (APC) and olfactory tubercle (OT, Panel C) and posterior piriform cortex (PPC, Panel E). The boxed regions in each panel are the zones depicted in Figs 3–6. OB = olfactory bulb, TT = tenia tecta. Panels B, D, and F are higher magnification views of the laminar structure of the areas. All zones have an outer molecular layer (layer 1). The AONpP has one cellular zone (layer 2), while the piriform cortices and OT have two (layers 2 and 3). Scale bar for B, D, and F = 200μm. See text for details.

Germinal zones that form neocortical projection cells (e.g., the “cortico-striatal junction” [27, 28]; broad regions of the developing telencephalon; [29]) also produce cells that migrate into regions of the olfactory cortex. Olfactory cortex is often defined as those areas receiving a direct projection from the olfactory bulb (OB: e.g., [30, 31]). These regions are, like the cerebral cortex, clearly laminated with a cell-poor outer molecular layer. However, they contain fewer layers [e.g., pars principalis of the anterior olfactory nucleus (AONpP) has 2 layers, while the anterior and posterior piriform cortices (APC, PPC) and olfactory tubercle (OT) have 3, Fig 2]. The AONpP, APC and PPC contain stereotypical pyramidal neurons but the OT does not [32, 33]. The purpose of the present work was to determine a) if neurons in olfactory cortical regions express the same transcription factors as cells in neocortical layers, b) if both superficial and deep markers are apparent and c) if the markers are uniformly distributed in all the regions. The presence of the markers would suggest that similar developmental and evolutionary processes shaped the different cortices.

## Materials and Methods

### Animals

These studies were carried out in strict accordance with the recommendations in the Guide for the Care and Use of Laboratory Animals of the National Institutes of Health. All protocols were approved by the University of Virginia’s Institutional Animal Care and Use Committee (PHS service assurance number #A3245-01; USDA registration #: 52-R-0011). All surgery was performed in aseptic conditions with isoflurane anesthesia, and all efforts were made to minimize suffering. Animals were euthanized by deep anesthesia with sodium pentobarbital (Euthasol, 0.39mg drug/gm body weight). All mice (C57Bl/6J Jackson Labs; Bar Harbor, ME) were housed in standard polypropylene cages with food (8604, Harlan, Frederick, MD) and water *ad libitum*. The colony was maintained on a 12:12 light:dark cycle in a temperature- and humidity-controlled room.

### Immunostaining and analysis

Fluorescence immunohistochemistry was used to stain free-floating 60 μm-thick vibratome sections. Mice (20–25 days old) were perfused with freshly prepared paraformaldehyde and allowed to postfix for 2 hrs at 4°C. The sections were rinsed 4 times in 0.01 M phosphate buffered saline (PBS pH 7.4). Next, the tissue was incubated in 0.01 M citrate buffer pH 8.5 at 80°C (2 X 15 min, [34]). After cooling at room temperature for 5 min, the sections were washed in PBS (2 X 2.5 min), permeablized in 0.03% Triton in PBS (TW: 4 X 5 min), and placed into blocking solution (0.5% normal donkey serum in TW; Jackson ImmunoResearch,West Grove PA) for one hr. Sections were then placed into primary antibody (Table 1) at least overnight at 4°C. They were then washed (PBS 4X5 minutes) and incubated in secondary antibody (1/250 to 1/450 in TW: Jackson ImmunoResearch donkey anti-rabbit: catalog number 711-165-152 or 711-545-152; donkey anti-goat: 705-165-147 or 705-545-147; donkey anti-mouse: 715-485-150 or 715-545-151; donkey anti-rat: 712-165-153) for 1 hr, washed again (PBS 4 X 5 min) and mounted on slides with SlowFade mounting media (Invitrogen: S36937). To observe tissue organization some sections were also Nissl-stained (640 nm Neurotrace; Invitrogen: N-21483). In each case, deletion of the primary antibody resulted in no staining.

**Table 1:** Antibodies.

Antigen	Cortical Layer	Immunogen	Manufacturer	Cat./lot #	Species	Dilution
NURR1 (NR4A2)	Subplate	Peptide mapping at the C-terminus of Nurr1of rat origin	Santa Cruz (Santa Cruz, CA)	SC-990/A0614	Rabbit Polyclonal	1/1000
TBR1	I II/III Vb VI SP	Amino acids 1–200 at the N-terminus of mouse Tbr1	Santa Cruz (Santa Cruz, CA)	SC-48816/10409	Rabbit Polyclonal	1/100
FOXP2	VI	Recombinant Protein Epitope Signature Tag (PrEST) antigen sequence: AQQLVFQQQLLQMQQLQQQQHLLSLQRQGLISIPPGQAALPVQSLPQAGLSPAEIQQLWKEVTGVHSMEDNGIKHGGLDLTTNNSSSTTSSNTSKASPPITHHS	Atlas Antibodies (Stockholm,Sweden)	HPA000382/ A38809	Rabbit Polyclonal	1/200
NOR1 (Nr4a3)	VI	Synthetic peptide conjugated to KLH derived from within residues 200–300 of Human Nr4a3	Abcam (Cambridge, MA)	Ab94507/ GR83936-2	Rabbit Polyclonal	1/500
CTIP2	Vb VI	Fusion protein between amino acids 1–150 of Ctip2	Abcam (Cambridge, MA)	Ab18465/ GR137972-21	Rat Monoclonal	1/1000
DARPP-32	VI	Purified Bovine DARPP-32	H. C. Hemmings, (Cornell University)	N/A	Mouse Monoclonal	
BRN2	II/III Vb	Synthetic peptide conjugated to KLH derived from within residues 1–100 of human Brn2	Abcam (Cambridge, MA)	Ab-94977/ GR54045-3	Rabbit polyclonal	1/100
CUX1	II/III IV	Epitope corresponding to amino acids 1111–1332 mapping at the C-terminus of CDP of mouse origin	Santa Cruz Biotechnology, Inc (Santa Cruz, CA)	SC-13024/ F2513	Rabbit Polyclonal	1/50
CART	Deep II/III	Rat Cart Amino Acid sequence: IPIYEKKYGQVPMCDAGEQCAVRKGARIGKLCDCPRGTSCNSFLLKCL[Disulfide bonds between C1–C3, C2–C5, C4–C6]	Phoenix Pharmaceuticals (Burlingame, CA)	H-003-62/ 01251–6	Rabbit Polyclonal	1/1000

### Cell Birthdating

To examine differences in the time of origins of neuronal classes pregnant mice were injected with 5-ethynyl-2'-deoxyuridine (EDU: Carbosynth, San Diego CA; 50 mg/kg). Two injections were administered separated by 2 hrs during the first hours of the lights-on period of either E12 or E16 (day of mating = E0). Brains from P20 subjects were sectioned and stained as above and EDU-containing cells visualized with ClickIt assay kits from Life Technologies (Grand Island, NY).

### Retrograde neuronal labeling

Iontophoretic injections of biotinylated dextran amines (BDA, 3000MW, Invitrogen, Carlsbad CA: D7135, Lot 57319A) were used to visualize the somas of identified projection neurons. Young (20–25 day old) mice were anesthetized with isoflurane and craniotomized under aseptic conditions. Glass micropipettes (tip size 20–50 μm) were lowered into the rostral half of the OB to ensure that injections would not overlap with the AON. BDA iontophoresed for 10 minutes (10% solution in citrate buffer: 10–30 μA positive current, 7 second duty cycle). After a 5 min incorporation period pipettes were slowly withdrawn and the wounds closed with surgical adhesive. Four to seven days later mice were deeply anesthetized with sodium pentobarbital (Euthasol, 0.39mg drug/gm body weight) and perfused transcardially with PBS followed by 4% buffered formaldehyde freshly depolymerized from paraformaldehyde. Vibratome sections of the brains were treated as above to assess the transcription factors expressed in BDA labeled cells in the AONpP, APC and PPC. Inspection of the injection sites indicated that the method labeled about 20–40% of the OB and verified that BDA did not encroach on structures in the peduncle

### Data Acquisition

In order to facilitate comparisons coronal sections were selected from standardized locations (Fig 2). The same area of the AON used in previous work [35, 36] was chosen: the region in the caudal olfactory peduncle where AONpP completely encircles the SVZ/ALAC core and layer 2 is still separate from the overlying cerebral cortex. The OT and APC were examined in rostral sections where the corpus callosum had thinned into a flat band dorsally and surrounded the anterior extent of the caudate/putamen. Sections of the PPC approximately 100 μm caudal to the crossing of the anterior commissure were selected. A series of horizontal sections were also examined for most of the antigens. Several sections from at least two subjects were examined for each of the markers and analyses outlined below. Images were collected at 20X with an Olympus confocal microscope. For each image two optical sections separated by 3 μm were combined, and montages were produced by tiling the images. Figures were produced by minimally adjusting the brightness and contrast of the images with Adobe Photoshop (San Jose, CA).

## Results

Fig 1a–1g shows the staining patterns for each of the probes used in the present study in the dorsal frontal neocortex. While the antibody to NURR1 marked cells in the neocortical subplate (Fig 1a; {11,12]), no staining was observed in any of the olfactory cortical regions, findings consistent with others suggesting no preplate exists in piriform regions during early life [30]. Immunostaining for TBR1 reliably marked both deep and superficial layer glutamatergic projection neurons (Fig 1b). Antibodies to NOR1, DAARP-32, FOXP2 and CTIP2 (Fig 1c and 1d) preferentially labeled deep (layers 5–6) pyramidal cells. BRN2 identified projection neurons in layers 2 and 3 as well as a small number of cells in layer 5 (Fig 1e). The superficial markers used included CUX1 (layer 2–4, Fig 1f) and CART (layer 3, Fig 1g). Patterns of staining with these markers in the four olfactory areas examined are detailed below.

### AONpP

**Fig 3:**
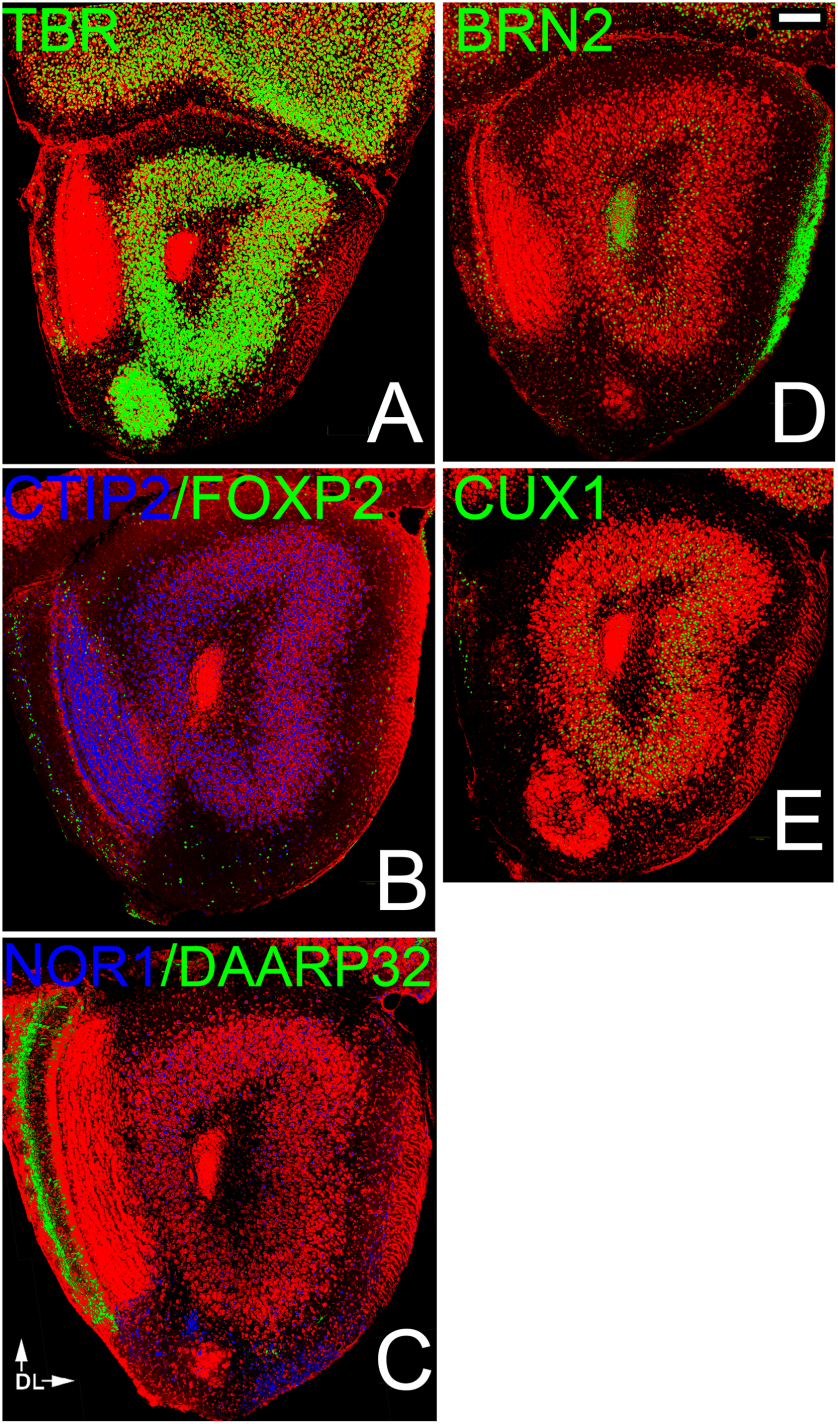
Patterns of neocortical layer markers in the AONpP. A). TBR1-labelled cells were found throughout Layer 2 of the AONpP as well as in the tenia tecta and mitral cell layer of the OB. B, C) Deep markers were differentially distributed in the region. Layer 2 exhibited dense and evenly-spread CTIP2-positive cells (Fig 3b), while NOR1 was found primarily in the dorsal portion of the structure (Fig 3c, top) Cells expressing the other two marker were rare and found primarily in layer 1: DARRP-32 (note dense staining in the glomerular layer of the OB at left, an area containing large numbers of dopaminergic interneurons, Fig 3c; Liu et al, 2013) and FOXP2 (most often found near the OB, Fig 3b). CTIP2 stained cells were also found in layer 1 but never in cells that expressed one of the other markers. The superficial markers were also differentially distributed. Both BRN2 (Fig 3d) and CUX1 (Fig 3e) were observed primarily in deep cells (except in pars medialis, where CUX1-labeled cells spanned the entire region) with highest densities in the region under the LOT (pars lateralis). All CUX1 cells also expressed BRN2, and over 90% of CUX1 and BRN2 cells also expressed CTIP2. The anti-BRN2 antibody also labeled the LOT (right) and RMS (core of the olfactory peduncle). Scale bar = 200μm. Dorsal to top, lateral to right.

AONpP is often divided into subregions (pars dorsalis, pars lateralis, pars ventroposterior and pars medialis) based on differences in organization and projections [37–38]. The cellular layer (layer 2) of AONpP encircles the central white matter of the olfactory peduncle and the subventricular region/rostral migratory stream (Fig 2a). The superficial plexiform region (layer 1, Fig 2b) envelopes layer 2 and underlies the LOT which is found on the lateral side of the peduncle. Layer 1 can be divided into layer 1a, containing axon collaterals from OB projection neurons and the apical dendrites of layer 2 neurons, and layer 1b, containing association axons from olfactory cortical regions and sparse interneurons. TBR1 immunopositive cells were widely spread throughout layer II as well as in the ventral tenia tecta (Fig 3a). The marker was absent from the internal granule cell layer in the OB, a region densely populated by GABAergic granule cells.

While all four of the deep cortical markers were found in AONpP, each was distributed differently. For example, layer 2 exhibited dense and evenly-distributed CTIP2-positive cells (Fig 3b), while NOR1 was found primarily in dorso-medial regions (pars dorsalis; Fig 3c) Only occasional, scattered DARRP-32 cells were observed in AONpP, though dense staining was observed in the glomerular layer of the OB, an area containing large numbers of dopaminergic interneurons (Fig 3c; [39]). In layer 1 scattered FOXP2 and CTIP2 (Fig 3b) positive cells were observed although never in the same cells. FOXP2 cells were most often found near the OB, while the sparse CTIP2 cells were more evenly distributed.

Superficial markers were also observed to have a differential distribution. Both BRN2 (Fig 3d) and CUX1 (Fig 3e) were observed primarily in deep cells (except in pars medialis, where CUX1-labeled cells spanned the entire region) with highest densities in the region under the LOT (pars lateralis). All CUX1 cells also expressed BRN2, and over 90% of CUX1 and BRN2 cells also expressed CTIP2. No CART staining was observed. The anti-BRN2 antibody also labeled the LOT and the rostral migratory stream/subventricular zone in the center of the olfactory peduncle [35].

### APC/PPC

**Fig 4:**
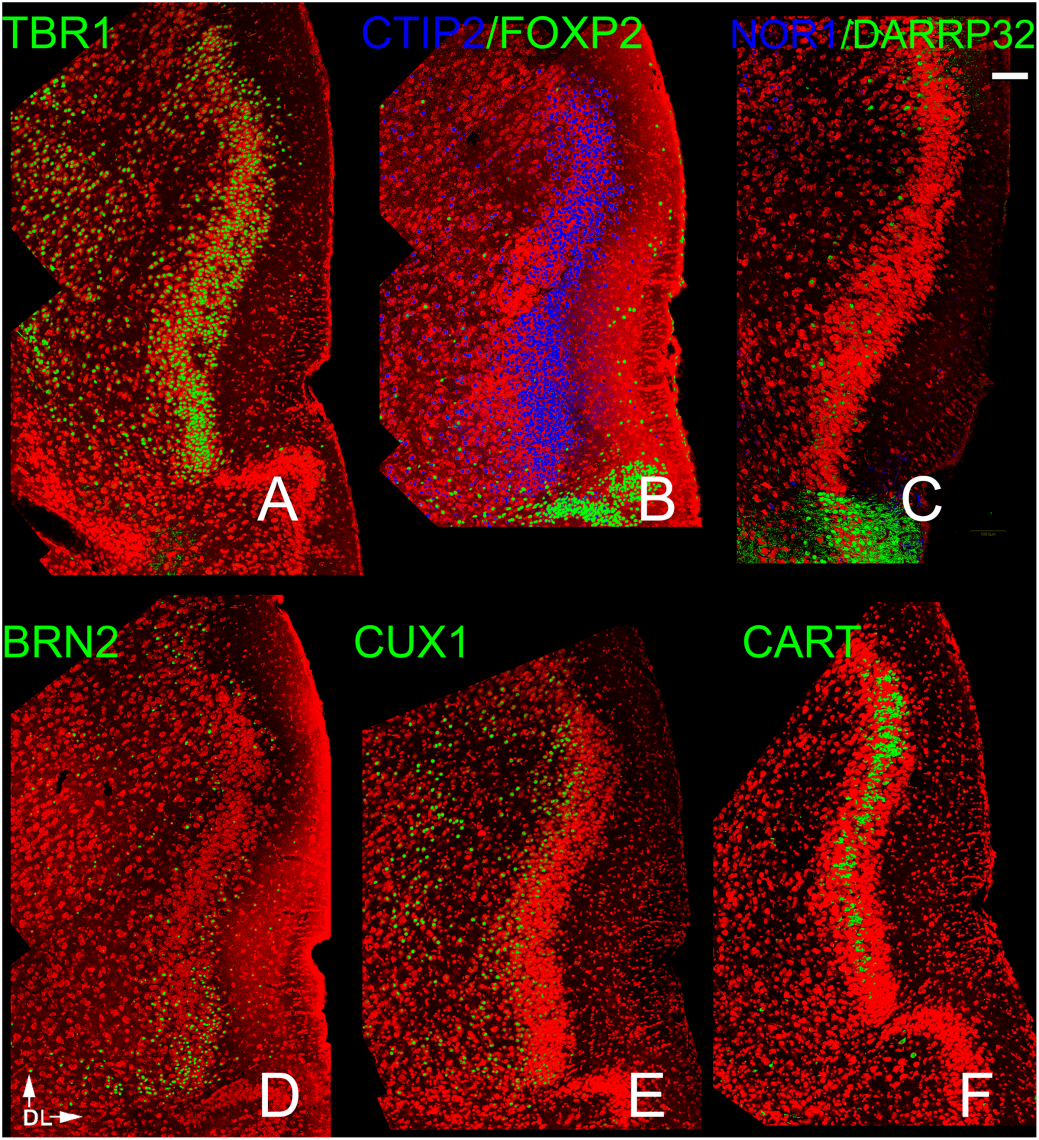
Patterns of neocortical layer markers in the APC. A) TBR-1 heavily labeled cells in Layer 2 as well as scattered cells in Layer 3. Of the 4 deep layer markers (B,C), only CTIP2 exhibited dense staining. The other three (FOXP2, NOR1 and DAARP32) labeled sparse number in Layers 1–3. The dense staining for FOXP2 and DAARP32 seen at the bottom of the figures sharply demarcates the APC from the more ventral OT. The other three makers exhibited very different patterns: BRN2 staining was found more in the ventral APC (D), CUX 1 in the deeper portions of both Layer 2 and 3 (E), and CART in the middle of Layer 2(F). Scale bar = 200μm. Dorsal to top, lateral to right.

**Fig 5:**
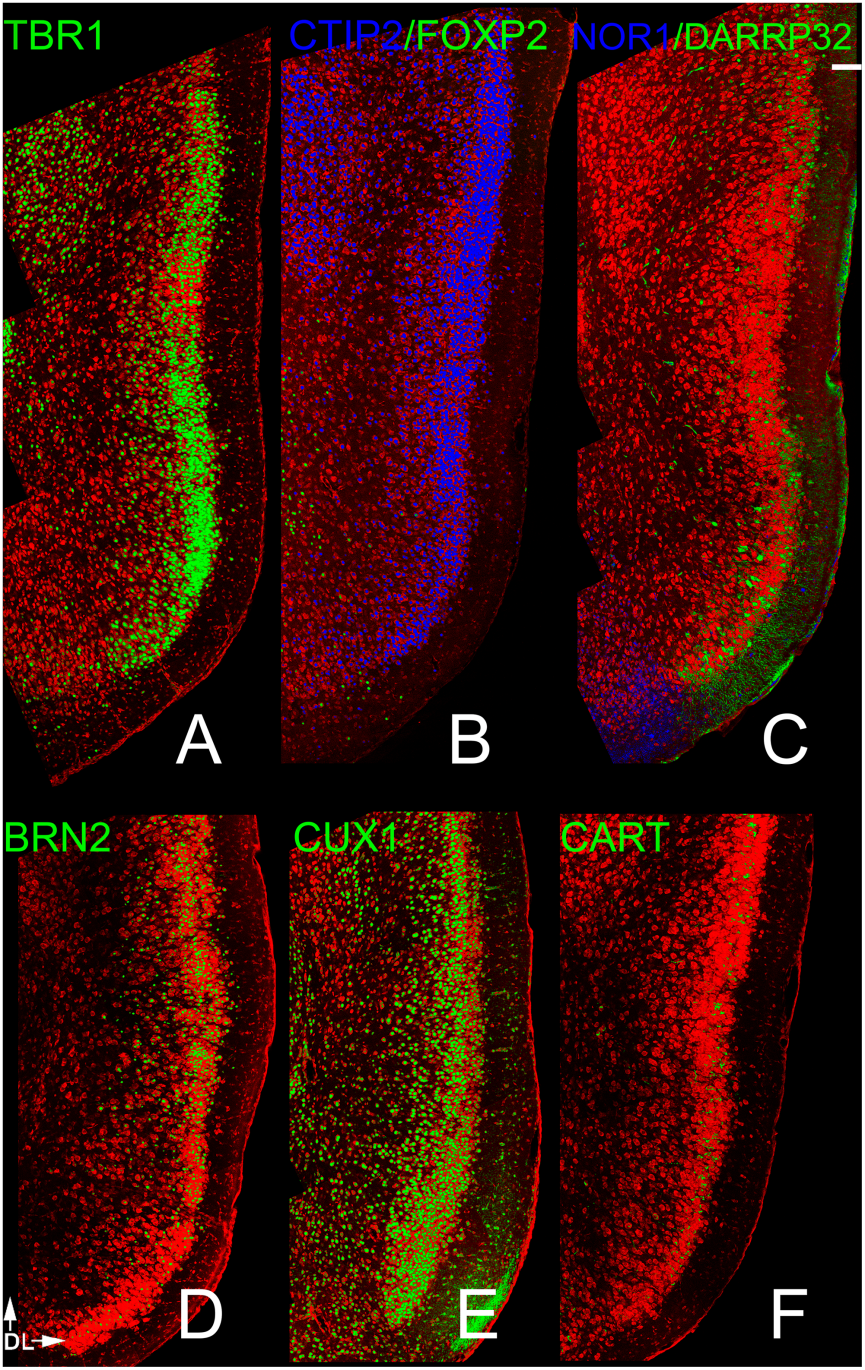
Patterns of neocortical layer markers in the PPC. A) TBR-1 heavily labeled cells in Layer 2 as well as scattered cells in Layer 3. As in the APC many cells in layers 2 and 3 exhibited the deep marker CTIP2 (B). Only widely scattered cells exhibited FOXP2 and DAARP 32 and NOR1 (B,C). The other three makers exhibited very different patterns: CUX 1 staining (E) was strong throughout layers 2 and 3, BRN2 staining much more modest in the same regions, CART was restricted to the middle of layer 2 (F). Scale bar = 200μm. Dorsal to top, lateral to right.

Three layers are generally recognized in the piriform cortex (PC; Fig 2c and 2e). Like AONpP it contains a similarly-organized superficial plexiform region (layer 1) encircling the LOT (Fig 2d). Layers 2 and 3 are both cellular regions. Layer 2 is thin and densely packed while layer 3 is broader with more scattered cells; both areas contain pyramidal neurons that were visualized with antibodies to TBR1 (Figs 4a and 5a). All three layers contain a variety of interneurons [40–43]. The APC has been subdivided into several different zones [31, 44] and differs from the PPC both on the basis of anatomy (e.g., APC provides more commissural projections, has a thinner layer 3 and receives a stronger input from the OB, [40,44] and function (e.g., the APC is considered to be more involved in odor identification, and the PPC in judging odor importance [45,46].

While both deep and superficial neocortical markers were found in the two regions, patterns differed both between the two areas and between these regions and the AONpP. All four deep markers were expressed. CTIP2-labeled cells were found throughout layer 2 in the PPC (Fig 5b), but were absent from the deep border of the region in the APC (Fig 4b). The other three markers were only sparsely observed and showed regional differences. For example, very small numbers of NOR1 cells were observed primarily in ventral portions layer 1 in both APC and PPC (Figs 4c and 5c). FOXP2 cells were only observed in APC sections, where they were most numerous in layer 1 and occasionally observed in layer 2. DARRP32 cells were found in layers 2 and 3 in both the APC and PPC. In sections of the PPC the marker was strongly expressed in the lateral OT; the sharp difference in staining delineated a clear boundary between the two regions (Figs 4c and 5c).

All three superficial markers were also observed in both regions. In the APC most cell bodies expressing BRN2 were observed in the ventral portion of the structure near the OT (Fig 4d). In the remaining portion of the APC, BRN2+ cells were concentrated in the superficial regions of both layer 2 and 3. In the PPC, BRN2 cells were evenly scattered though the structure in both layers 2 and 3 (Fig 4d). CUX1 positive cells were observed in the deep portions of both layers 2 and 3 of the APC (Fig 4e). In the PPC they were found predominately in deep layer 2, but were more widely scatted in Layer 3 (Fig 5e). In both regions, many though not all CUX1 positive cells in layer 2 colocalized with CTIP2, but only a subpopulation of those in layer 3 exhibited both markers. Not all CTIP2 cells expressed CUX1 though, especially in the superficial portion of layer 2. CART immunoreactivity was observed in a discrete band in the middle of layer 2 in both the APC and PPC (Figs 4f and 5f).

### Olfactory Tubercle

Found adjacent to the APC on the ventromedial side of the brain (Fig 2c), the OT has a thin layer 1 (Fig 2f). The OT has a dense layer 2 that forms a series of gyrations: the apical peaks of these gyrations contain small sets of granule cells. The deep layer 3 includes several kinds of projection and intrinsic neurons [32], the island of Calleja [47] and elements of the ventral pallidum and nucleus accumbens [32, 33, 48].

**Fig 6:**
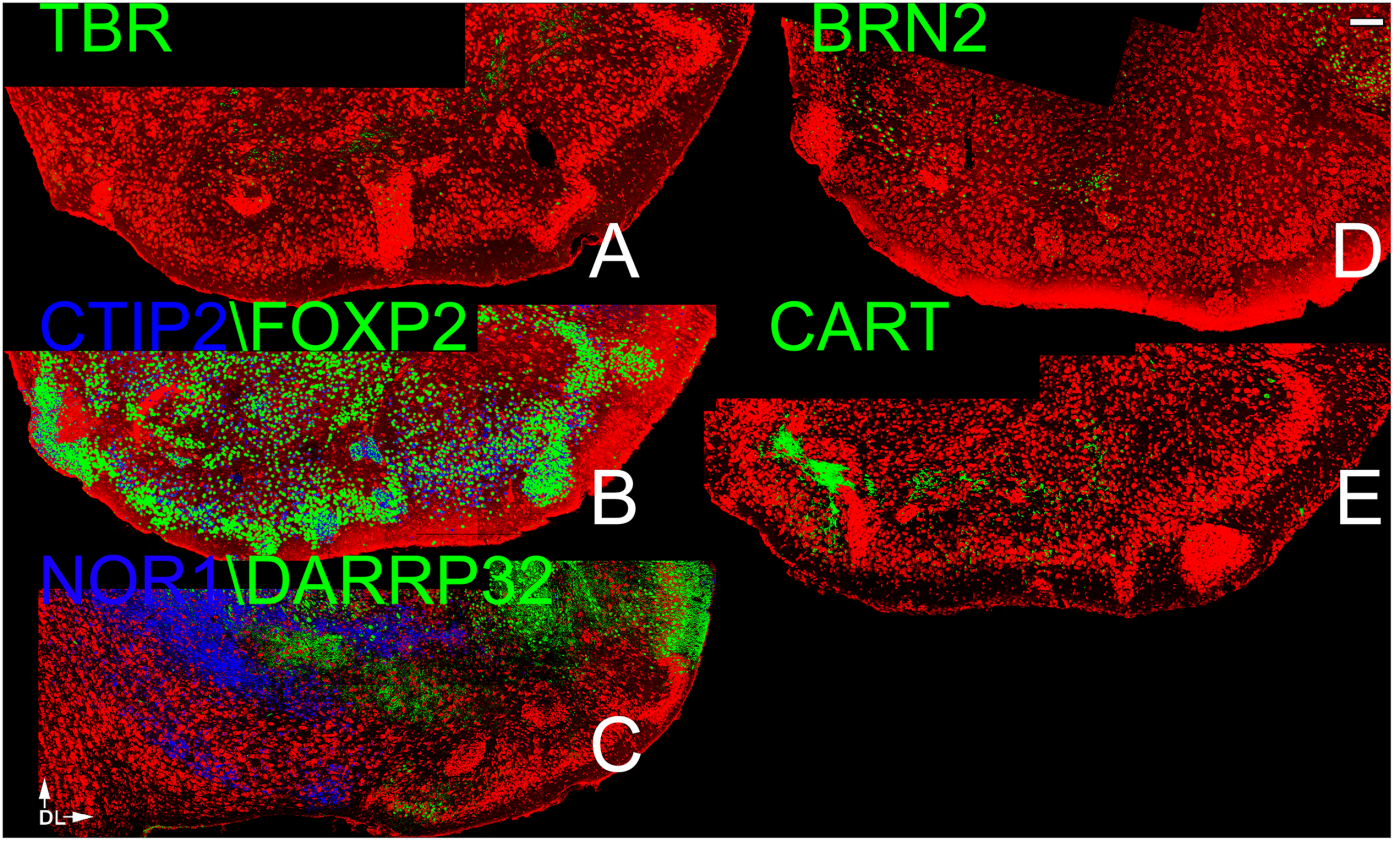
Patterns of neocortical layer markers in the OT. A) TBR-1 (A), BRN2 (D) and CART (E) were only found scattered in the very deepest regions of the OT. All four of the deep laminar markers heavily labeled the region. Both CTIP2 and FOXP2 cells were broadly present in Layer 2 and scattered in Layer 3 (Fig 6b). On the medial side most FOXP2 cells coexpressed CTIP2 but the percentage of cells with both markers was reduced laterally. DARRP-32 cells were dense on the lateral side near the APC and in deep regions of the OT, while NOR1 cells were found in Layer 2 in the medial OT (Fig 6c). Scale bar = 200μm. Dorsal to top, lateral to right.

TBR1 immunoreactive cells were not observed in the region (Fig 6a). Both CTIP2 and FOXP2 cells were broadly present in layer 2 and scattered in layer 3 (Fig 6b). On the medial side most FOXP2 cells coexpressed CTIP2 but the percentage of cells with both markers was reduced laterally. DARRP-32 cells were dense on the lateral side near the APC and in deep regions of the OT, while NOR1 cells were found in layer 2 in the medial OT (Fig 6c). Sparse cells exhibiting the superficial markers BRN2 and CART were found in the very deep portions of the region (Fig 6d and 6e).

### Cell Birthdating

**Fig 7:**
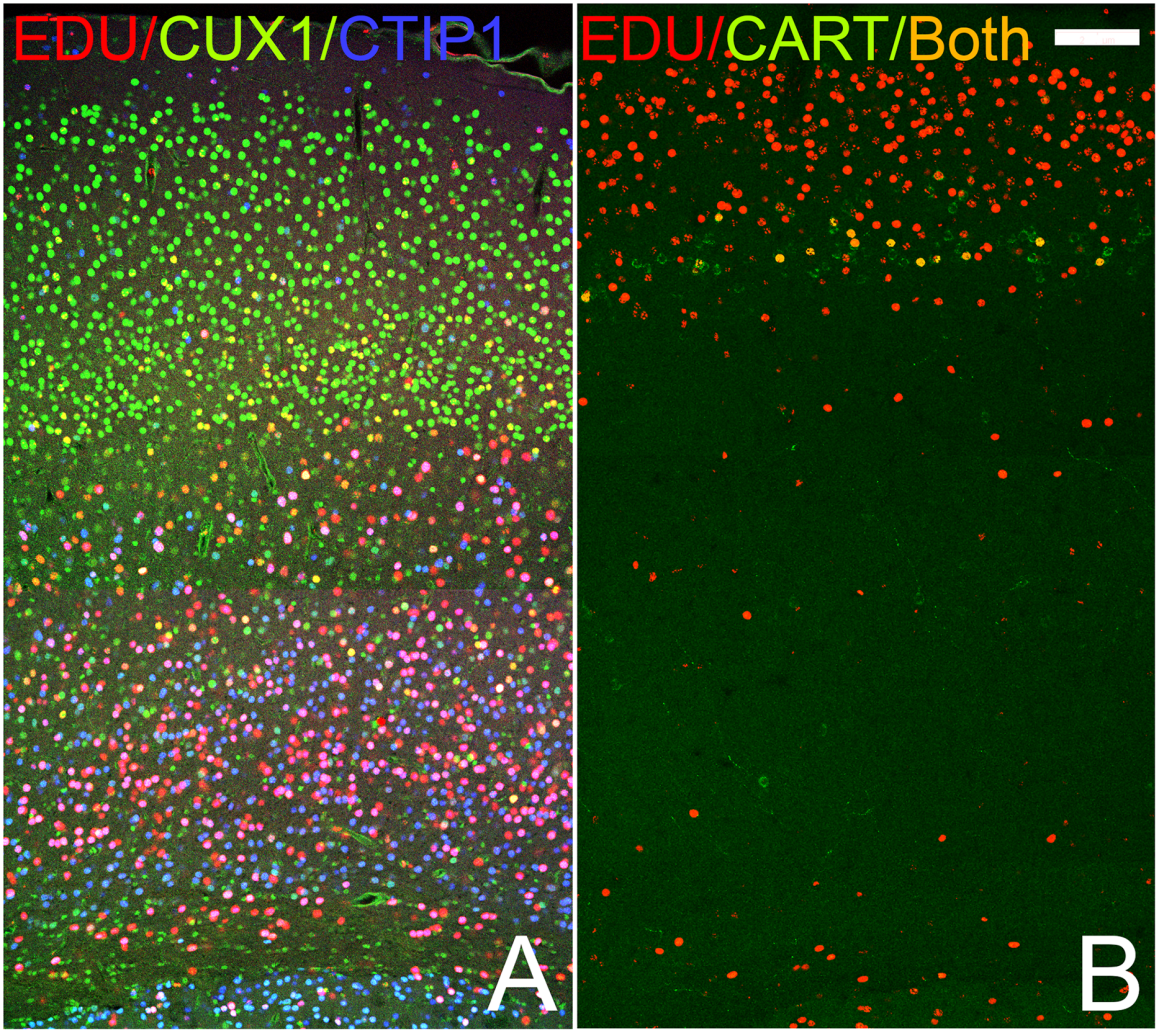
Patterns of staining in the frontal cerebral cortex of pups receiving the mitotic marker EDU on either E12 (A) or E16 (B). Note the age-related differences in EDU (red) staining: at E12 EDU is found primarily deep in the cortex (bottom of panel), while at E16 it is found in superficial layers (top). In the E12 tissue (A) substantial numbers of cells expressing the deep marker CTIP2 co-labelled with EDU (pink) while only a few of the cells labelled with CUX also have the maker (yellow). E16 injections (B) labeled cells in layer 3 expressing the superficial marker CART as wells as more superficial cells. The marker labels all proliferating cells, including glial and perivascular cells. Scale bar = 200μm.

In order to determine if olfactory cortical cells expressing different markers have birthdays similar to those in the neocortex, the mitotic marker EDU was injected in pregnant dams on either E12 or E16 (during the period of deep or superficial neocortical cell production, respectively: [1,2,9]) and subsequently tissue from labeled P20 pups was examined. Fig 7 demonstrates that these injections did indeed target different neocortical markers. For example, after E12 injections, cells labeled with EDU were found primarily in the deep cerebral cortex and preferentially in cells expressing the deep marker CTIP2 compared to the superficial marker CUX1 (Fig 7a). E16 treatment tagged cells in the layer 2–3 of the cerebral cortex including cells that expressed CART (Fig 7b). It is important to note that since E12 is very early in cortical maturation, labelled precursor cells at this stage will undergo more subsequent mitotic cycles and thus produce a larger and more diverse cohort of labeled cells that than cells incorporating EDU at E16. In addition, the marker labels all proliferating cells, including glial and perivascular cells.

**Fig 8:**
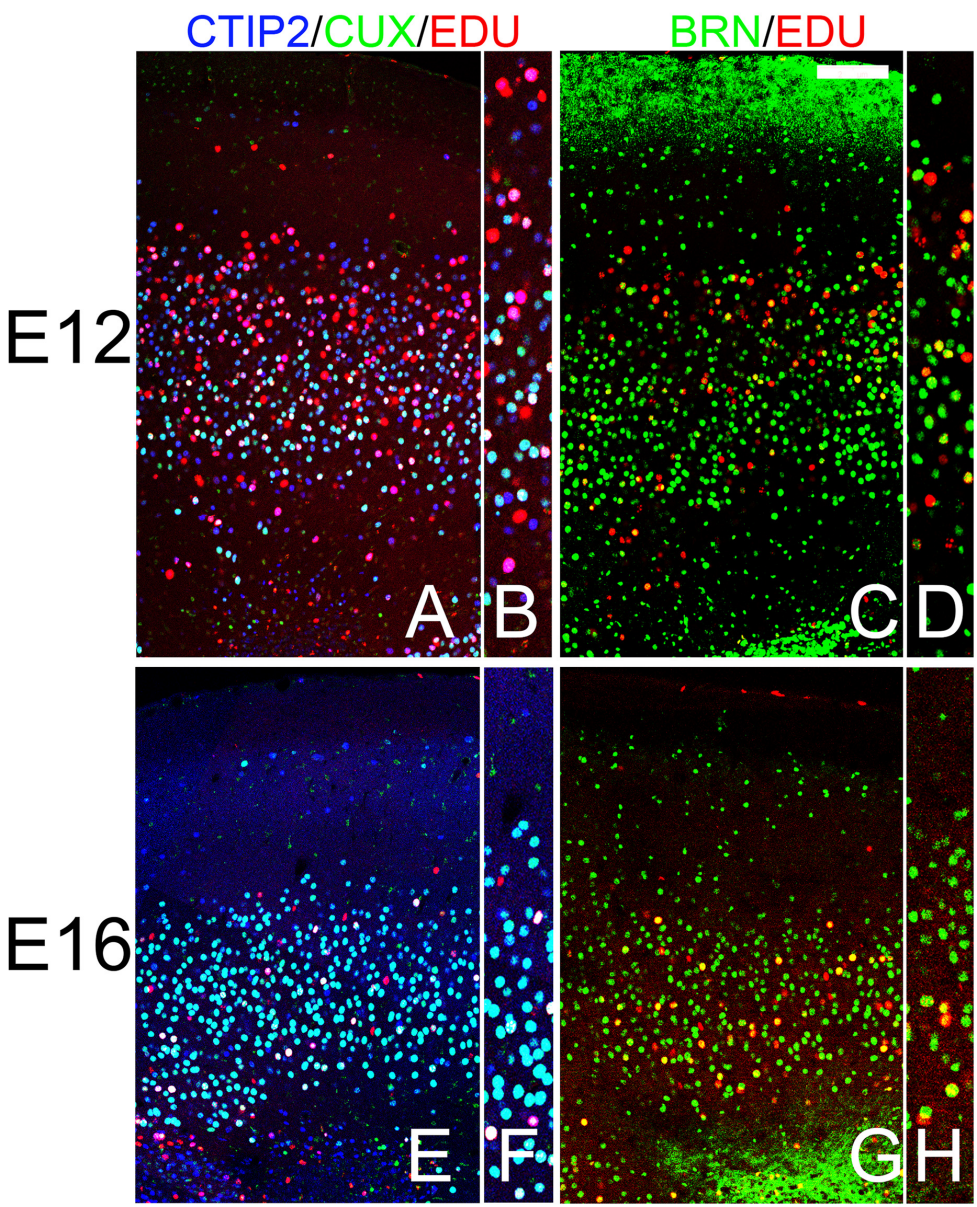
Patterns of staining in the AONpP of pups that receiving the mitotic marker EDU either on E12 (A-D) or E16 (E-H). Panels B, D, F and H provide higher power views of the cellular regions of the figures to their left. A gradient of EDU labeling was observed with more cells in the superficial half of layer 2 labeled at E12 and more in the deep portion at E16. In the E12 samples both CUX1/CTIP2 (light blue; CUX1/CTIP2+EDU are depicted in white) and CTIP2 alone cells (CTIP2+EDU depicted as pink) were found co-labeled with EDU (Fig 8A and 8B) and a small number of BRN2/EDU-positive cells (yellow) were also observed (Fig 8C and 8D), mostly in the superficial portion of layer 2. By E16, a) EDU was rarely found in CTIP2 single-labeled cells (Fig 8E and 8F) and b) BRN2/EDU cells were much more abundant (Fig 8G and 8H). Taken together, the findings suggest that the pattern of cell birth is consistent with that seen in the cerebral cortex in that cells with cortical “deep markers” were labelled early while those with “superficial” markers were produced on E16. Scale bar = 200μm.

In the AONpP of animals labeled at E12, EDU was observed throughout layer 2 with approximately twice as many labeled cells found in the outer half of the cellular zone as in the inner region. By E16 the difference reversed: the number of labeled cell observed in the deeper portion of the layer was double that of the superficial zone (compare Fig 8a and 8c with 8b and 8d). These findings are consistent with previous observations of an “outside-in” pattern of histogenesis in the region [49]. In the E12 samples both CUX1/CTIP2 and CTIP2 alone cells were found co-labeled with EDU (Fig 8a) and a small number of BRN2/EDU-positive cell were also observed (Fig 8c), mostly in the deep portion of layer 2. By E16, a) EDU was never found in CTIP2 single-labeled cells (Fig 8b) and b) BRN2/EDU cells were much more abundant (Fig 8d). Taken together, the findings suggest that the pattern of cell birth is consistent with that seen in the cerebral cortex in that cells with cortical “deep markers” were labelled early while those with “superficial” markers were produced on E16.

**Fig 9:**
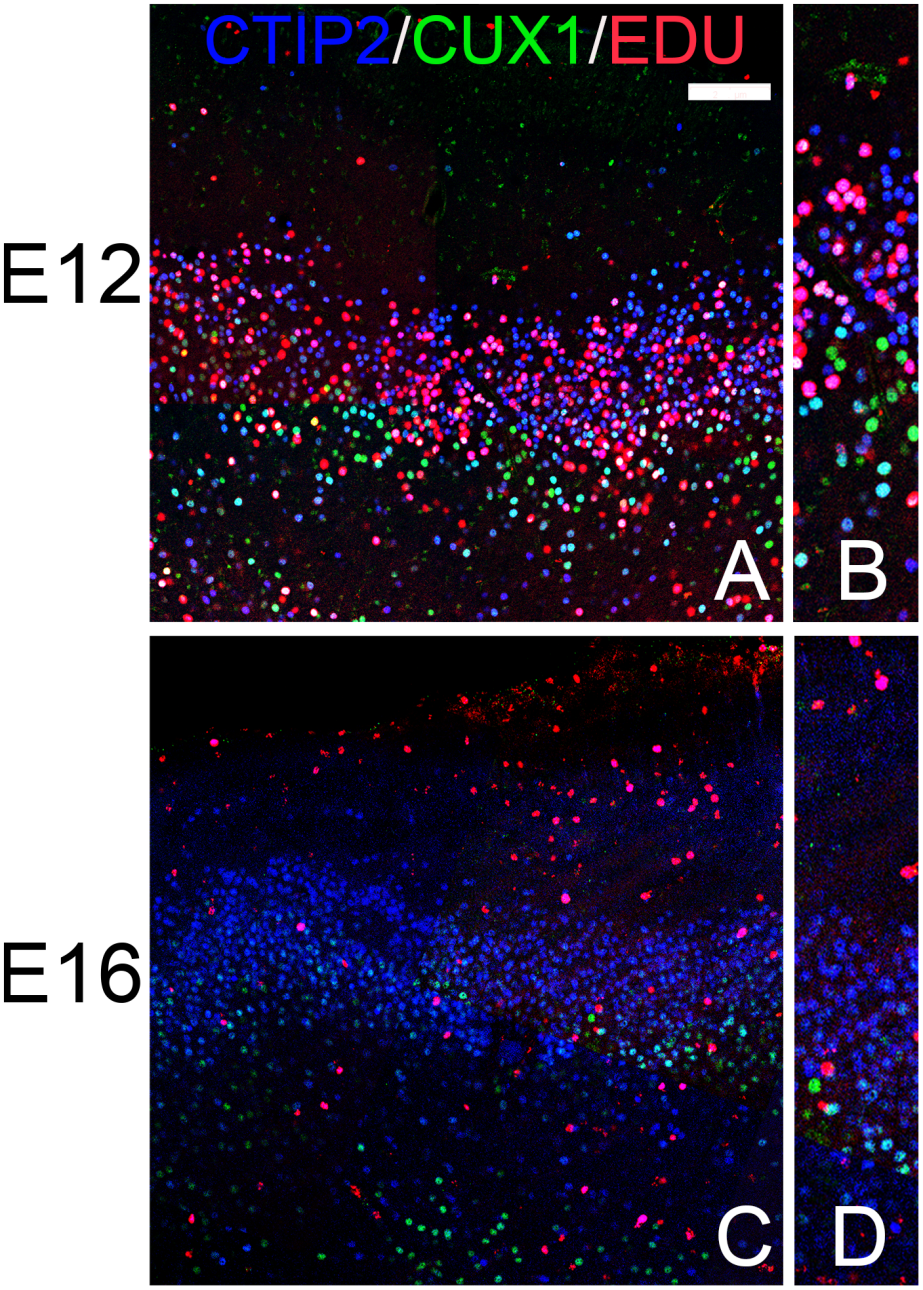
Patterns of staining in the APC of pups that receiving the mitotic marker EDU either on E12 (A,B) or E16 (C,D). Panels B and D provide higher power views of the cellular regions of the figures to their left. E12 injections labeled many cells in Layer 3 and in the deep portion of Layer 2. CUX1/CTIP2 (light blue; CUX1/CTIP2+EDU are depicted in white) and CTIP2+EDU (pink) were observed. Few labelled cells were observed in layers 2 and 3 after E16 EDU treatment. Scale bar = 200μm.

**Fig 10:**
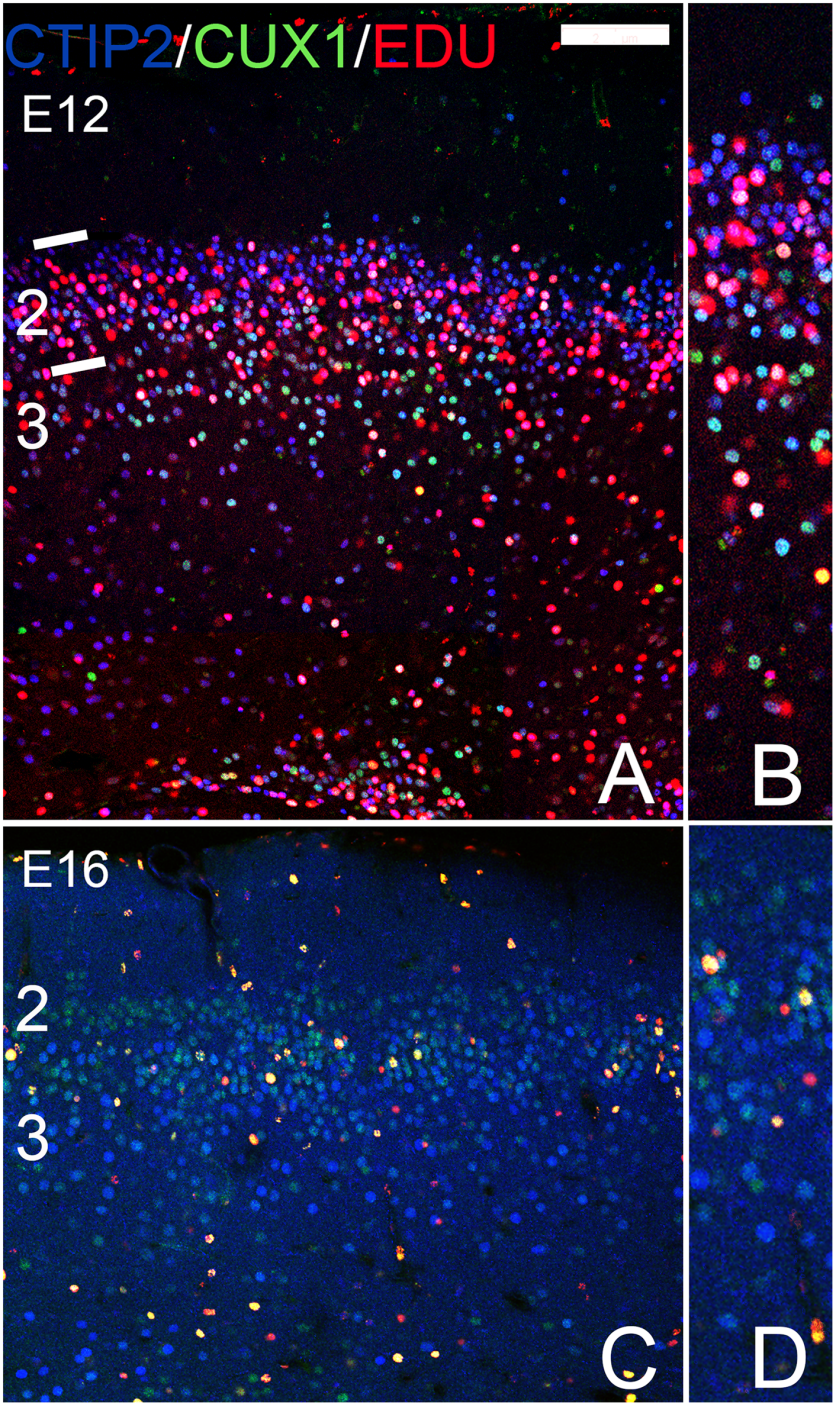
Patterns of staining in the PPC of mice that receiving the mitotic marker EDU either on E12 (A,B) or E16 (C,D). Panels B and D provide higher power views of the cellular regions of the figures to their left. The E12 injection labeled many CTIP2 cells (CTIP2+EDU depicted as pink) in layer 3 and in the deep portion of Layer 2. Relatively few CUX1 +EDU (yellow) or CUX1/CTIP2+EDU (white) were observed. Scattered labelled cells were seen in layer 2 after EDU injection on E16; most were CUX1+EDU (yellow) positive. Numbers and lines on the left margin of A and C delineate cortical layers. Scale bar = 200μm.

In the APC of mice labeled at E12, EDU-positive cells were widely distributed across layers 2 and 3 (Fig 9a). CTIP2/EDU and BRN2/EDU double-labelled cells were apparent but few CUX1 or CART cells also contained EDU. Labeled cells in subjects from the E16 EDU group were relative rare in the region (Fig 9b) though BRN2, CUX1 and CART cells with EDU were occasionally observed. The PPC sections examined exhibited obvious deep-to-superficial pattern of development consistent with previous reports [50]: in E12 labelled subjects more EDU-positive cells were found in deep areas while in E 16 they were mostly in superficial layer 2 (Fig 10a and 10b). E12 subjects exhibited many CTIP2/EDU cells but only a few CTIP2/CUX1/EDU. By E16 more CTIP2/CUX1/EDU cells were observed as well as cells labeled with the CART antibody. Taken together, these finding suggest that markers found early or late in the cerebral cortex appear in a similar sequence the APC and PPC.

The OT was not examined as closely as most of these markers were not observed in the region. General gradients of cell proliferation described by Bayer [51] were observed (e.g., E12 subjects had more labeled figures both deep and laterally than those from the E16 subjects), and more EDU positive cells that expressed either CTIP2 or FOXP2 were observed in the earlier injection group.

### BDA Injections

**Fig 11:**
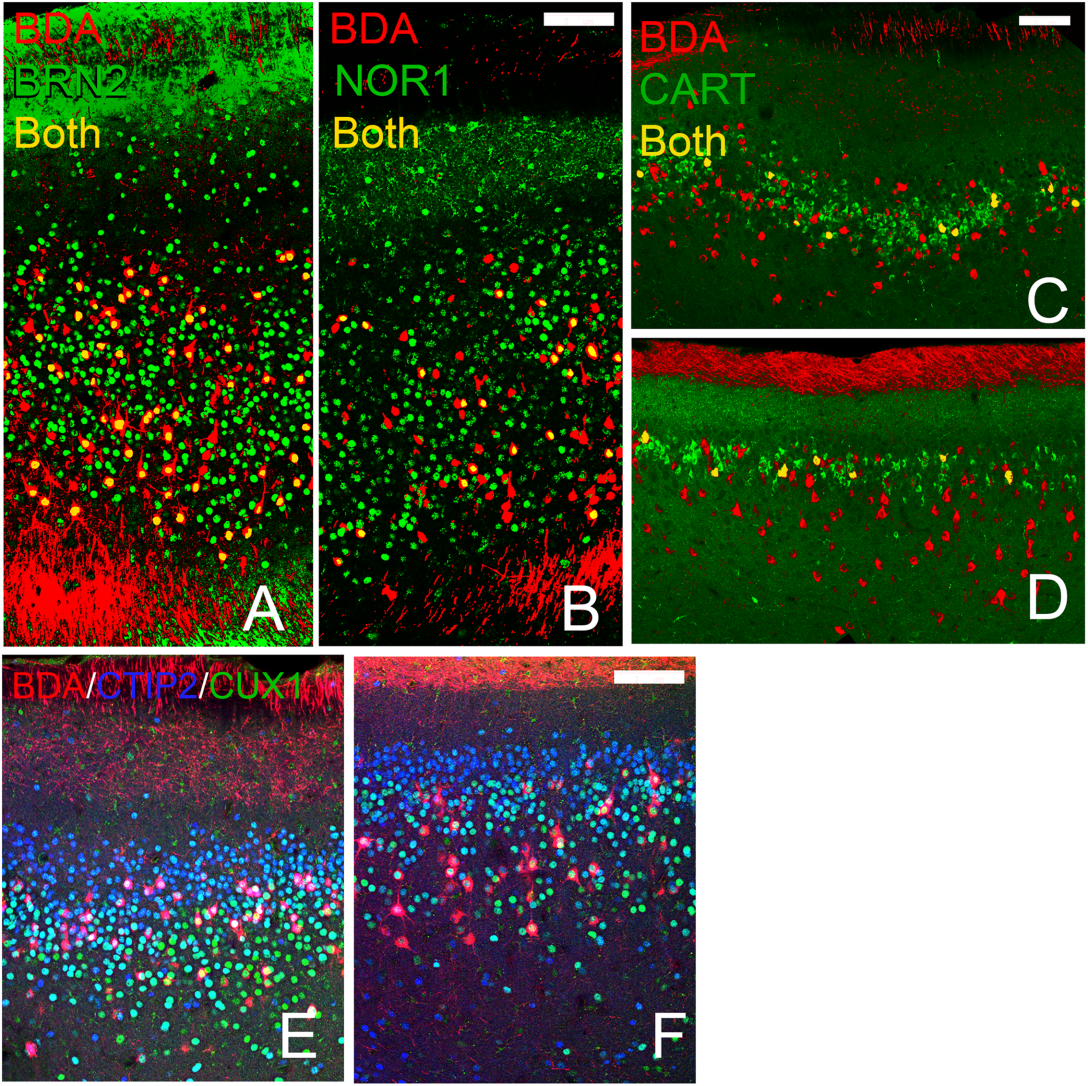
Retrograde tracer (BDA, red) injections into the OB labelled cell bodies expressing each of the deep and superficial markers tested. This figure depicts examples for each structure projects to the OB. A, B: AONpP sections exhibiting double labeled cells (yellow) containing BDA and BRN2 (A) and NOR1 (B). Piriform cortices: double labeling for CART (APC: C, PPC: D, yellow), CUX1 (APC: E, PPC:F yellow) and CTIP2 (APC: E, PPC:F pink). White cells in panels E and F are triple labeled for BDA, CUX1 and CTIP2. Scale bars = 200μm.

As mentioned, the antigens examined above mark neocortical neurons with different synaptic targets; for example, deep cells project out of the cerebral cortex while superficial cells make predominately intracortical connections. Each of the olfactory regions studied in the present work also innervate numerous regions [31]. The differential staining observed above might therefore represent different classes of projection neurons. In order to gauge this possibility a retrograde tracer was injected into the OB as each area except the OT has back-projections to the bulb. Retrogradely-labeled cells expressing all of the deep or superficial markers observed in each of the three region (AON: CTIP2, NOR1, BRN2, and CUX1; APC and PPC: CTIP2, DARPP32, BRN2, CUX1, and CART, Fig 11) except for FOXP2. The latter result is understandable as FOXP2 cells were rarely observed in the main, pyramidal cell containing layers of the areas studied.

## Discussion

The results outlined above indicate that cells in all of the olfactory regions examined express transcription factors that are found both deep and superficially in the developing neocortex. The pattern of expression of markers was complicated and varied both between and within the areas studied as indicated in Table 2.

**Table 2:** Regional distribution of neocortical markers in olfactory cortex.

	NURR1	TBR1	FOXP2	CTIP2	DARRP-32	NOR1	BRN2	CUX1	CART
	*Subplate*	*Plate*	*DEEP*					*Superficial*	
**AONpP**									
Layer 1		rare	scattered dorsomedial, ventromedial	scattered	rare	+	scattered	rare	
Layer 2		+++	rare	+++		++ higher dorsally	deep: low medially	deep except medially	
**APC**									
Layer 1		rare	scattered	scattered			rare		
Layer 2		+++		+++ superficial	scattered		+ higher ventral	deep	middle
Layer 3		++	rare	++	rare	rare	scattered	++	
**PPC**									
Layer 1		rare		scattered	rare		rare	scattered	
Layer 2		+++		+++	scattered		+	+++	middle
Layer 3		+++	rare	+++	rare		+	+++	
**OT**									
Layer 1			scattered	rare	+++ near APC				
Layer 2			+++	++	occasional island				
Layer 3			++	+	rare		scattered		scattered

+++: very dense;

++: dense;

+ light;

cell empty: absent

The cerebral cortex is formed from cells arising from both the early cortical subplate and the later cortical plate. Nissl studies have not found evidence of a subplate in olfactory regions [30], and results described above indicate that none of the areas examined above had cells immunoreactive to the subplate marker NURR1. However, since NURR1 is only expressed in a subset of preplate neurons [12], the existence of a subplate contribution cannot be ruled out.

TBR1 is a marker for the projection neurons derived from the pallium, including neocortical, hippocampal, AONpP, APC and PPC pyramidal cells (Figs 1b, 3a, 4a and 5a). The marker, however, was not observed in the OT. Though formally listed as an as a portion of the “olfactory cortex” (e.g., [30]), the OT does not derive from the pallium but from the subpallium (e.g., [52–54]) and is both embyrologically and functionally related to the ventral striatum. Several lines of evidence suggest that the OT is organized differently from other “cortical” structures: it does not have pyramidal shaped output neurons [32] and it lacks both the radial organization [55, 56] and the association fiber system seen in other cortices [33]. Bayer [51] reported that patterns of early cell proliferation in the OT are not deep-to-superficial as seen in the neocortex but more similar to the gradients seen in the globus pallidus and substantia innominata, and indeed a large literature indicates that the region is an extension of the basal ganglia [48,57]. The results presented above add more evidence: few markers seen in the neocortex were observed in the OT and those that were highly expressed (e.g., CTIP2 and FOXP2) seamlessly merged with similar cells in the overlying striatum.

There are several views as to the developmental origins of the remaining areas. Studies examining patterns of very early gene expression suggest that the regions arise differently. In this view pyramidal neurons that reside in the AONpP originate in the ventral pallium, which also produces the TBR1-expressing mitral/tufted cells of the OB (and may contribute cells in ventral portions of the piriform cortex and claustrum, as well as in portions of the lateral amygdala [15, 52, 53, 58]. In contrast, the lateral pallium sources neurons to the piriform cortices through germinal zones found near the cortico-striatal junction (and may also contribute cells to the dorsal claustrum and the basolateral amygdala [15, 28, 52, 53, 59]. However, Garcia-Moreno et al. [29], tracing the movement of cells in labeled in young embryos, did not observe this specificity. They reported that many broad regions of the developing telencephalon contribute cells to both the PC and OT, though they did not specifically examine the cellular origins of the AONpP. Nevertheless, the data described above indicates that there are differences in the expression of cortical markers between the AONpP and PC. For example, all three regions expressed the deep cortical marker CTIP2, but NOR1 was expressed much more in the AONpP, while there were more DAARP32 cells in the APC and PPC. In all regions the superficial markers BRN2 and CUX1 were expressed, but CART was only observed in the APC and PPC. Taken together, these observations highlight the fact the AON is a not just a simpler or less laminated version of the piriform cortex, but a distinct neural region [38].

Along with differences between these regions, variations within the structures were also observed (Table 2). For example, the deep layer marker CTIP2 was found throughout the cell layer of the AONpP while NOR1 was found primarily in dorsal regions. CTIP2-labeled cells were found throughout layer 2 in the PPC, but were absent from the deep border of the region in the APC. Superficial markers exhibited the same diversity. In the AONpP, CUX1 positive cells were observed primarily in deep cells except in the ventromedial region, where they spanned layer 2. In the APC, BRN2-expressing neurons were observed in the ventral portion of the structure or in the superficial portions of both layer 2 and 3, while in the PPC BRN2 cells were evenly scattered though the structure in both layers 2 and 3. CART immunoreactivity was observed in a discrete band in the middle of Layer 2 in both the APC and PPC. These results suggest that the cellular layers of the olfactory cortex consist of a variable mosaic of neurons of different origins, and as such that they are more complicated than often portrayed.

Neocortical neurons found in different layers arise on different days. For example, in mice, cells destined for layer 6 are born about E12 while layer 2–3 neurons emerge 3–4 days later [1, 2, 9]. It was important to determine whether the cells in the olfactory cortices that express superficial vs deep markers exhibited similar patterns of development. Cell proliferation sequences vary dramatically across the olfactory regions. For example, Sarma et al. [60] reported that cell production in the piriform cortex of mice is similar to that of the neocortex, with the bulk of pyramidal cells forming from E12–14 and continued cell production through E16. Bayer [50] indicated cells destined for the piriform cortex are generated before those for the AON in rats and that both regions exhibit caudal-to-rostral gradients, but that the PC forms from deep-to-superficially, while in the AONpP the gradient is reversed with superficial cells added before deep ones. Results presented above are consistent these observations: a deep-to-superficial pattern of labeled cells was observed in the APC and PPC (Figs 9 and 10) and a superficial-to-deep pattern in the AONpP (Fig 8). The expression of deep and superficial neocortical laminar markers within these gradients was similar to those seen in the cerebral cortex. For example, cells expressing CTIP2 alone, a deep maker, were labeled only after early (E12) EDU injections, while markers found in regions of the superficial neocortex (e.g., CUX1, and CART) were more abundant after injections at E16.

Harris and Shepherd [61] have suggested that one way to view commonalties across the cerebral cortex is to focus on the developmental precursor populations. They argued that cells with similar backgrounds follow homologous genetic programs resulting in the cells assuming similar synaptic connectivity even in different circuits. Neocortical neurons expressing different transcription factors project to different targets. For example, layer 6 cells preferentially innervate thalamic targets while layer 2–3 cells form mostly intracortical connections. Perhaps a similar pattern exists in the areas examined here: neurons expressing the same transcription factors in different regions may play similar roles in different olfactory circuits. The areas examined are widely interconnected and have synaptic relations with many other targets as well (e.g., [31]). An exhaustive catalog would difficult to produce, so we began by looking at a target to which three of the four regions (AONpP, APC and PPC) project, the backprojection to the OB. Injections of retrograde tracers into the OB labeled cells that expressed each of the markers examined in all three structures, indicating that all species of neurons participate in the pathway, and perhaps suggesting little specificity. Nevertheless, it is possible that different cells projecting to the same target might provide different inputs: for example, subpopulations of axons might target different neuronal elements within the OB or relay different channels of information to the same neurons [43, 62].

While the data presented above suggests that similar rules govern the development of the neocortex and olfactory cortex, several caveats are apparent. The first revolves around defining a “labeled cell”. The fluorescent immunostaining methods employed resulted in a continuum of labeling from very light to quite intense. Since setting a criterion level solely on the basis of fluorescence intensity without other information would be arbitrary, in the present work any signal detected was considered above threshold. Second, some of the laminar markers are expressed in many regions other than the developing neocortex so their presence in a tissue does not definitively define a cell’s developmental origins. For example, both DAARRP-32 and CART are expressed in brain regions involved in reward pathways (the striatum, [63] and hypothalamus; [64] respectively). Examples of this phenomenon are also apparent in the present work. BRN2 was observed both the rostral migratory stream/subventricular layer and in the lateral olfactory tract (Fig 3d); regions with very few mature neurons or synapses, and the markers seen in the OT (Fig 6) are expressed both in striatal and cortical tissues. Therefore, while the results presented above are consistent with the notion that olfactory and neocortical projection neurons arise in a similar fashion, more detailed work examining the exact points of origin and routes of migration of the olfactory projection neurons might prove otherwise.

The results presented above indicate that the neocortex, AONpP, APC, and PPC exhibit similar temporal changes in cell genesis and that in each area neurons establish their residence in gradients based on their time of origin. How many other ways are the tissues similar? Comparisons of the structure and evolution of forebrain cortices has prompted decades of research and theorizing (e.g., [65–67]). According to classical evolutionary theory the cerebral and olfactory cortices derive from different portions of the developing dorsal telencephalon. One view is that originally their organization was similar and that they have diverged as they evolved. Evidence for the hypothesis includes observations that, like most of the olfactory regions, the reptilian cerebral cortex has three layers (outer and inner plexiform layers sandwiching a cellular layer), and pyramidal shaped projection neurons (e.g., [66, 68, 69]). With the emergence of mammals, the neocortex elaborated to include more superficial layers due to the addition of intermediate progenitor cells in the subventricular zone (e.g., [70,71]). However, another view suggests that the basic cell types and circuitry has been around for a very long time, that the synaptic circuits of all the cortices are fundamentally similar (e.g., [72]), and that in mammals this same organization has been simply elaborated by lamination (e.g., [67, 73,74]). The data presented above suggests that olfactory cortices do not simply contain elements found in the deep layers of the cerebral cortex, as CART and CUX1, markers found in superficial neocortical zones, were observed in the AONpP, AC and PPC. Furthermore, the findings indicate that the AONpP, APC and PPC of mice are different from each other and differ in local patterns even within structures. The findings suggest that the olfactory cortices are not merely remnant architypes of the primordial forebrain but varied, independent regions. Further research comparing the regions should lend insight into both the evolutionary and developmental sequence by which forebrain cortices arise.
